# Novel Cruzain Inhibitors for the Treatment of Chagas’ Disease

**DOI:** 10.1111/j.1747-0285.2012.01416.x

**Published:** 2012-09

**Authors:** Kathleen E Rogers, Henrik Keränen, Jacob D Durrant, Joseline Ratnam, Allison Doak, Michelle R Arkin, J Andrew McCammon

**Affiliations:** 1 Biomedical Sciences Graduate Program, University of California San DiegoLa Jolla, CA 92093, USA; 2 Department of Cell and Molecular Biology, Uppsala UniversityS-751 24 Uppsala, Sweden; 3 Department of Chemistry and Biochemistry, University of California San DiegoLa Jolla, CA 92093, USA; 4 Department of Pharmaceutical Chemistry, University of California San FranciscoSan Francisco, CA 94158, USA; 5 Department of Chemistry and Biochemistry, NSF Center for Theoretical Biological Physics, National Biomedical Computation Resource, University of California San DiegoLa Jolla, CA 92093, USA; 6 Department of Pharmacology, University of California San DiegoLa Jolla, CA 92093, USA; 7 Howard Hughes Medical Institute, University of California San DiegoLa Jolla, CA, USA

**Keywords:** Chagas’ disease, computer-aided drug discovery, cruzain, cruzipain, cysteine protease inhibitor, *Trypanosoma cruzi*

## Abstract

The protozoan parasite *Trypanosoma cruzi*, the etiological agent of Chagas’ disease, affects millions of individuals and continues to be an important global health concern. The poor efficacy and unfavorable side effects of current treatments necessitate novel therapeutics. Cruzain, the major cysteine protease of *T. cruzi*, is one potential novel target. Recent advances in a class of vinyl sulfone inhibitors are encouraging; however, as most potential therapeutics fail in clinical trials and both disease progression and resistance call for combination therapy with several drugs, the identification of additional classes of inhibitory molecules is essential. Using an exhaustive virtual-screening and experimental validation approach, we identify several additional small-molecule cruzain inhibitors. Further optimization of these chemical scaffolds could lead to the development of novel drugs useful in the treatment of Chagas’ disease.

The World Health Organization estimates that over 10 million people are infected by the protozoan parasite *Trypanosoma cruzi*, with another 25 million at risk^a^. The associated illness, called Chagas’ disease, is spread through a triatomine vector or through blood transfusion (1,2). The acute phase lasts at most a few months and is characterized by mild symptoms such as fever, malaise, facial edema, generalized lymphadenopathy, and hepatosplenomegaly. In approximately 30% of infected patients, parasite multiplication *via* asynchronous cycles contributes to the chronic stage of the disease, with the associated cell destruction, reinfection within the reticuloendothelial system, and organ infection (3). Infection of the heart, digestive tract, and central nervous system can lead to fatal heart-rhythm abnormalities, megacolon, and dementia, respectively (4,5).

*Trypanosoma cruzi* is not susceptible to many of the drugs used to treat closely related parasites like *Trypanosoma brucei.* Benznidazole and nifurtimox are the only available therapies for acute-phase Chagas’ disease. These nitroheterocyclics are highly toxic and have poor efficacy in long-lasting chronic infections (6–8). No extensive studies of the long-term sequellae of these therapeutics have been conducted in humans, but several reports of neuropathy and tumorigenic or carcinogenic effects have been described (6,7). Efforts to develop a vaccine against *T. cruzi* have also failed thus far, likely because the disease pathology has an autoimmune component (9).

The major *T. cruzi* cysteine proteinase cruzain (also referred to as cruzipain, the full-length native enzyme) has been shown to be crucial for all stages of the parasite life cycle. This papain-like cysteine protease is thought to play an important role in differentiation, cell invasion, intracellular multiplication, and immune evasion (10,11). Furthermore, studies have demonstrated that cysteine proteinase inhibitors have trypanocidal activity with negligible mammalian toxicity (12).

Previous efforts have identified vinyl sulfones, sulfonates, and sulfonamides as high-affinity cruzain inhibitors (13,14); one of these vinyl sulfones, K11777, is currently undergoing Investigational New Drug enabling studies (15,16). α-ketoamide-, α-ketoacid, α-ketoester-, aldehyde-, and ketone-based inhibitors have also been described (17–19). While these successes are encouraging, many potential drugs, including those that enter clinical trials, ultimately fail to gain approval (20), and those that are approved are subject to growing parasitic resistance. Consequently, a diverse set of inhibitory scaffolds that can be optimized into distinct therapeutic candidates is urgently needed.

Hoping to contribute to this ever-growing diverse set of compounds, we here use an advanced virtual-screening methodology that accounts for receptor flexibility to identify three promising non-covalent inhibitors of *T. cruzi* cruzain.

## Experimental Methods

### Ligand preparation

A small-molecule library was prepared from the ligands of the NCI Diversity Set II using the Schrödinger ligprep program^b^. Protonation states were assigned at pH 5.5 to mimic the natural acidic environment of the *T. cruzi* digestive vacuole. Multiple tautomers and stereoisomers were generated. One ligand could not be processed by ligprep; instead, Discovery Studio^c^ was used to add hydrogen atoms to this ligand and to optimize its geometry.

### Initial screen against the crystal structure

The prepared ligand models of this small-molecule library were docked into a 1.20 Å crystal structure of cruzain (PDB ID: 1ME4) (18), with hydrogen atoms included using PDB2PQR (21,22) at pH 5.5. Residues CYS25 and H159 (called H162 by some) formed the thiolate–imidazolium pair required for the catalytic mechanism (23) of the proteinase at this pH. This initial virtual screen was performed using the cdocker docking software^c^ with a docking sphere 15 Å in diameter centered on the coordinates of the crystallographic ligand.

### Rescoring protocol

The cdocker-predicted pose of each ligand model was rescored using six additional scoring functions: LigScore1, LigScore2 (24), PLP1, PLP2 (25), PMF (26), and PMF04 (27). The best-scoring models as evaluated using each of these seven scoring functions were compiled into a new small-molecule library of 302 models (182 unique ligands) enriched for predicted cruzain inhibitors.

### Molecular dynamics simulations

The molecular dynamics simulations used in the current study have been described previously (28). In brief, the simulations were based on a 1.20 Å cruzain crystal structure (PDB ID: 1ME4) (18) protonated at pH 5.5 to mimic the natural acidic environment of the *T. cruzi* digestive vacuole. Following appropriate minimization and equilibration, five distinct 20-ns simulations of the cruzain protein bound to a hydroxymethyl ketone inhibitor, [1-(1-BENZYL-3-HYDROXY-2-OXO-PROPYLCARBAMOYL)-2-PHENYL-ETHYL]-CARBAMIC ACID BENZYL ESTER, were performed. The *gromos* clustering algorithm (29) was used to cluster 4002 conformations extracted from the simulations every 50 fs. We found that decreasing the cutoff below 0.95 Å resulted in a precipitous rise in the number of clusters; consequently, we chose an RMSD cutoff of 0.95 Å, which yielded 24 clusters. The central member of each cluster, considered most representative, was selected for subsequent analysis; this set of central members is said to constitute an *ensemble*.

### Relaxed -complex screen

The 302 compound models of the enriched small-molecule library were docked into the 24 clusters of the ensemble using cdocker (Accelrys). Each of these docked small-molecule models was rescored with the following scoring functions: LigScore2 (24), PLP1, PLP2 (25), PMF (26), and PMF04 (27). For each ligand/scoring function pair, an ensemble-average score was calculated according to the following equation: 

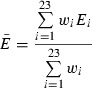
1
where 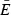 is the weighted ensemble-average score, *w*_*i*_ is the size of cluster *i*, and *E*_*i*_ is the best score of each unique ligand, independent of tautomeric or stereoisomeric form, docked into the centroid of cluster *i*.

Two methods were used to select compounds for subsequent experimental validation. First, for each of the five scoring functions, the compounds were ranked from best to worst by the ensemble-average score. The top seven compounds were selected from each of these five ordered lists and merged into a single list of potential binders. Second, the average rank of each compound across all five scoring functions was calculated. The compounds were then reordered by this average rank, and the top thirty were likewise identified as potential binders. Any compound indicated by either of these two protocols was subsequently recommended for preliminary experimental validation.

### Enzymatic assays

Each compound was obtained from the National Cancer Institute’s Development Therapeutics Program, which guaranteed 90% purity. Compounds were tested for cruzain enzymatic inhibition using a protocol that has been described previously (30). The eight compounds with the lowest IC_50_ values were assessed for aggregation by observing enzymatic activity under varying experimental conditions. As detergent is known to disrupt colloidal aggregation (31), inhibition in the absence of detergent was compared with inhibition in the presence of Triton X-100 (0.02%) and Tween (0.002%). The reducing agent was also varied (10 mm DTT or 10 mm Beta-Mercaptoethanol). Each experimental condition was tested in at least two separate experiments. Finally, a dynamic light scattering technique, described in detail elsewhere (32), was applied to the top four compounds to further confirm the presence or absence of aggregation. Additional experimental details can be found in the Supporting Information.

### Final pose predictions

All compounds submitted for experimental validation were subsequently docked a final time into the binding pocket of the crystal structure (PDB ID: 1ME4) using the Induced Fit Docking (IFD) module of the Maestro 9.2 (Schrödinger, LLC) computer package with Glide XP precision (33,34). For each of the top three nonaggregating ligands, the best-scoring pose that positioned the inhibitor near the crucial catalytic triad was selected and visualized using pymol^d^. Although the top poses using this IFD protocol were generally similar to those from the relaxed complex scheme (RCS) cdocker work, we choose to show them here in the commonly represented crystal structure conformation for ease of recognition to the reader of subsites within the well-characterized active site of cruzain.

## Results and Discussion

Discovered by Carlos Chagas in 1909 (35,36), *T. cruzi* is one of only two known pathogenic *Trypanosoma* species. Current trypanocidal therapeutics like nifurtimox and benznidazole are inadequate because they are toxic (6–8), subject to growing resistance (37), and ineffective at eradicating the parasite and preventing cardiomyopathy over the long term (38). Given the dire need for novel therapies, we here use virtual-screening methods to identify three promising inhibitors of cruzain, a critical cysteine protease required for *T. cruzi* survival.

### Weaknesses of virtual-screening

Virtual-screening techniques have been used to identify a number of inhibitors in recent years [see, for example, references (39–45)]. Though widely used, these screens are often characterized by many false positives and negatives. Two principal weaknesses explain these inaccuracies. First, there are errors intrinsic to the scoring functions themselves. Because virtual-screening efforts often attempt to identify true binders from among the many thousands of molecules in a compound library, they are generally optimized for speed at the expense of accuracy. Second, current docking programs account for, at best, only limited receptor flexibility. When a small-molecule binder encounters its receptor *in vivo*, that receptor often undergoes conformational rearrangements or an ‘induced fit’ to better accommodate the ligand. These *holo* conformations can differ significantly from those of x-ray crystallographic structures. Even if the perfect docking scoring function did exist, it could not accurately predict binding affinity if receptor flexibility were ignored. In the current work, we use a scheme designed to minimize both these sources of error.

### Overcoming inaccuracies inherent to the scoring functions themselves

The ligands of the NCI Diversity Set II^e^ were initially docked into a cruzain crystal structure using cdocker^c^ because this program was able to capture the crystallographic poses of two positive controls. Docking with AutoDock (46) was initially performed, but docked and crystallographic poses differed significantly. The cdocker-docked poses were subsequently rescored using several different scoring functions, and potential binders were selected by consensus rather than by the score of any single function. Consensus scoring has two advantages. First, when multiple scoring functions are combined, the errors of each may in part cancel out (47). Second, different scoring functions likely have different intrinsic weaknesses and strengths. Some, for example, may better account for hydrophobic contacts, while others better capture electrostatic interactions. When combined, accuracy may be improved if the weaknesses and strengths of the constituent functions are complementary (48).

The scoring functions used in the current work come from different classes and thus provide independent assessments of ligand binding that may be complementary. Scoring functions fall into one of three categories: force field, empirical, and knowledge-based. Force field scoring functions like that used by cdocker, based on the CHARMm force field, evaluate ligand binding by accounting for bonded and nonbonded atomic interactions explicitly. Empirical scoring functions like LigScore1, LigScore2, PLP1, and PLP2 are based on counting the number of different types of receptor-ligand interactions (e.g., hydrogen-bond and hydrophobic interactions) as well as countable changes in molecular properties (e.g., the number of rotatable bonds immobilized upon ligand binding). Finally, knowledge-based scoring functions like PMF and PMF04 are based on statistical analyses of large structural databases like subsets of the Protein Data Bank (49). Intermolecular interactions that occur more often than expected by pure chance are assumed to be energetically favorable.

The top candidate binders from this initial crystal structure screen, as judged by consensus scoring, were compiled into a single list of 302 small-molecule models that were subsequently subjected to further study.

### Overcoming inaccuracies caused by ignoring full receptor flexibility

While consensus scoring may have helped overcome in part the errors intrinsic to each individual scoring function, those caused by ignoring receptor flexibility remained. To overcome this second challenge, we first studied receptor dynamics by performing five distinct 20-ns molecular dynamics simulations of cruzain, described elsewhere (28). The protein conformations sampled during these simulations were clustered into 24 groups using an RMSD-based clustering algorithm (29). Each of the centroid members of each cluster, considered to be the most representative, was identified, and the group of all centroids is said to constitute an *ensemble*.

Having identified multiple cruzain conformations, we redocked the 302 small-molecule models identified in the initial crystal structure screen into each of the 24 members of the ensemble, again using cdocker. The ligands were then reranked by an ensemble-average score that was not dependent on a single crystal structure but rather accounted for receptor flexibility. This multireceptor docking protocol, called the RCS, has been used successfully in the past to identify inhibitors of FKBP (40), HIV integrase (39), and *T. brucei* RNA editing ligase 1 (41), among others (42–44).

### Final rescoring

As each of the 302 small-molecule models was docked into 24 different cruzain conformations, there were 7248 docked poses in all. Each of these 7248 poses was rescored, again using multiple scoring functions. Ensemble-average scores were calculated for each scoring function, and the 302 small-molecule models were appropriately ranked.

Two criteria were used to identify likely inhibitors from among these ranked compounds. First, we selected the seven best inhibitors as predicted by each of the individual scoring functions and merged them into a single list of 22 candidates. Second, the top 30 predicted ligands as judged by the ensemble-average rank were likewise identified. In all, 37 unique candidate inhibitors were selected; of these, the best 30 were tested experimentally.

### Predicted binding modes

**Figure 1:**
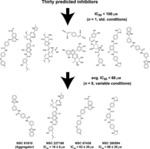
The structures of all experimentally validated compounds. Associated IC_50_ values are given for the top three non-aggregating inhibitors. Means and standard deviations were calculated by considering all IC_50_ values associated with each compound, measured under varying experimental conditions as described in the Experimental Methods (*n* = 8). ^*^NSC 61610 appears to inhibit cruzain nonspecifically via aggregation (see the Supporting Information).

Of the 30 compounds tested, eight inhibited cruzain at 100 μm (Figure 1, all structures below the first arrow). Using standard conditions, the best-scoring compound had an initial IC_50_ value of 471 nm (see Figure 1, asterisk). Subsequent experiments suggested that this compound inhibited cruzain non-specifically *via* aggregation (Supporting Information). Fortunately, three other compounds, although less potent, did in fact appear to inhibit cruzain specifically (Supporting Information). These three compounds, NSC 227186, NSC 67436, and NSC 260594, have IC_50_ values of 16, 63, and 66 μm, respectively (see Figure 1, bottom row). While these compounds lack the nanomolar affinity characteristic of approved drugs, they do possess the low micromolar affinity typical of lead compounds. With proper optimization, including fragment addition, moiety swapping, and similarity searching, these compounds could be transformed into viable drug candidates. We are hopeful that these new leads will be helpful to those in the drug discovery community targeting Chagas’ disease.

**Figure 2:**
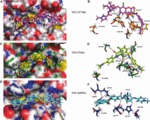
The predicted binding poses and receptor-ligand interactions of the experimentally validated inhibitors. A, C, and E: Compounds and cruzain active sites are shown in stick and surface representation, respectively. Active site domains are also labeled (S1–S3, S1′, S3′). The poses shown were obtained using the Induced Fit Docking module of the Schrödinger Maestro computer package. B, D, and F: The predicted receptor-ligand interactions, with hydrogen bonds represented as black dotted lines. Residues predicted to participate in receptor-ligand interactions, as well as the residues of the catalytic triad, are visualized.

Figure 2 shows the predicted binding poses (a, c, e) and important interactions (b, d, f) associated with each of the three most promising compounds using the IFD protocol and visualized in pymol (described in Experimental Methods). Figure 2B shows the predicted polar contacts of NSC 227186 with GLN19, THR185, and TRP184; TRP184 is also oriented for possible aromatic stacking with one of the ligand aromatic moieties. GLU208 (called GLU205 by some) is predicted to swing even further away from the cruzain S2 subsite than is seen in the 1ME4 crystal structure, allowing a ligand methylpyrrolidine moiety to occupy the site, reminiscent of the Phe and Tyr rings of several known inhibitors (13–18,50,51). An oxane moiety is also predicted to occupy the S3′ subsite (13).

NSC 67436 (Figure 2D) is predicted to form even more interactions with the cruzain receptor, including various hydrogen-bond interactions with residues GLN19, GLY66, ALA141, ASP161, and ASN182, as well as possible aromatic stacking with HIS162. Two distinct cyclohexanamine moieties are predicted to occupy the S2 and S1′ subsites, and a chlorocyclohexane (4-chlorocyclohexane-1,3-dicarbaldehyde) moiety is predicted to bind near S1. S4 and S3′ are also occupied, both with distinct imidazoline rings (13).

Figure 2F shows the hydrogen bonds predicted to form between NSC 260594 and MET68, ASN69, and ASP161. Again reminiscent of several known ligands, the S2 subsite is occupied by an aromatic ring system, and a slightly rotated GLU208 accommodates the larger aromatic moiety (1-methyl-6-nitro-decahydroquinolin-4-amine).

In an attempt to identify predicted binding characteristics that might aid the identification of future inhibitors, we used the IFD module of the Schrödinger Maestro computer package to re-dock the 30 experimentally tested compounds into a crystallographic active site model (PDB ID: 1ME4) at pH 5.5. Unlike cdocker docking, the IFD protocol allows for local active site conformational changes and so is judged to better predict ligand binding, albeit at the cost of speed. The most favorable IFD poses of the validated inhibitors (Figure 2) consistently placed ligand atoms near the catalytic triad in a position that could conceivably compromise cruzain enzymatic activity. Additionally, the IFD poses of these compounds also place at least one aromatic ring in the S2 subsite, a site known to be favorable for the binding of Phe and Tyr aromatic side chains (13–18,50,51).

These predicted binding poses are promising because they represent unique scaffolds that differ significantly from those of previously identified small-molecule inhibitors. While the poses included here are mere predictions, we are hoped that they will guide future optimization of these experimentally validated ligands.

## Conclusions

Chagas’ disease is the leading cause of heart failure in Latin America, affecting over 10 million individuals worldwide. Although progress has been made in the treatment of the disease, especially given the recent success of K11777, multiple cruzain inhibitors are needed given the difficulty of obtaining FDA approval and ever-progressing drug resistance. The work presented here provides chemical scaffolds that, with further optimization, could be developed into promising therapeutics for Chagas’ disease. The exhaustive virtual-screening approach and thorough experimental validation used to identify these leads also represents a promising and useful method for inhibitor identification.

## Notes

a WHO. Chagas Disease (American trypanosomiasis), Fact Sheet 340.

c Discovery Studio, version 2.1; Accelrys: San Diego, CA, 92121, U.S.A, 2008.

d The PyMOL Molecular Graphics System, Version 1.2r3pre, Schrödinger, LLC.

e The NCI/DTP Open Chemical Repository, http://dtp.cancer.gov.

## Supporting Information

Appendix S1 Description of experimental validation methods and assessment of non-specific inhibition via aggregation.

Figure S1 IC_50_ curves of each under various experimental conditions.

Table S1 Preliminary experimental validationresults for compounds with IC_50_ values of100 μMor lower.

## References

[1] Rassi A, Rassi A, Marin-Neto JA (2010). Chagas disease. Lancet.

[2] Shulman IA, Appleman MD, Saxena S, Hiti AL, Kirchhoff LV (1997). Specific antibodies to *Trypanosoma cruzi* among blood donors in Los Angeles, California. Transfusion.

[3] Higuchi MdeL, Benvenuti LA, Martins Reis M, Metzger M (2003). Pathophysiology of the heart in Chagas’ disease: current status and new developments. Cardiovasc Res.

[4] Tanowitz HB, Kirchhoff LV, Simon D, Morris SA, Weiss LM, Wittner M (1992). Chagas’ disease. Clin Microbiol Rev.

[5] Prata A (2001). Clinical and epidemiological aspects of Chagas disease. Lancet Infect Dis.

[6] Coura JR, de Castro SL (2002). A critical review on Chagas disease chemotherapy. Mem Inst Oswaldo Cruz.

[7] Castro JA, de Mecca MM, Bartel LC (2006). Toxic side effects of drugs used to treat Chagas’ disease (American trypanosomiasis). Hum Exp Toxicol.

[8] Jackson Y, Alirol E, Getaz L, Wolff H, Combescure C, Chappuis F (2010). Tolerance and safety of nifurtimox in patients with chronic chagas disease. Clin Infect Dis.

[9] Quijano-Hernandez I, Dumonteil E (2011). Advances and challenges towards a vaccine against Chagas disease. Hum Vaccin.

[10] Tomas AM, Miles MA, Kelly JM (1997). Overexpression of cruzipain, the major cysteine proteinase of *Trypanosoma cruzi*, is associated with enhanced metacyclogenesis. Eur J Biochem.

[11] Doyle PS, Zhou YM, Hsieh I, Greenbaum DC, McKerrow JH, Engel JC (2011). The *Trypanosoma cruzi* protease cruzain mediates immune evasion. PLoS Pathog.

[12] McKerrow JH, Rosenthal PJ, Swenerton R, Doyle P (2008). Development of protease inhibitors for protozoan infections. Curr Opin Infect Dis.

[13] Brinen LS, Hansell E, Cheng J, Roush WR, McKerrow JH, Fletterick RJ (2000). A target within the target: probing cruzain’s P1′ site to define structural determinants for the Chagas’ disease protease. Structure.

[14] Kerr ID, Lee JH, Farady CJ, Marion R, Rickert M, Sajid M, Pandey KC, Caffrey CR, Legac J, Hansell E, McKerrow JH, Craik CS, Rosenthal PJ, Brinen LS (2009). Vinyl sulfones as antiparasitic agents and a structural basis for drug design. J Biol Chem.

[15] Bryant C, Kerr ID, Debnath M, Ang KK, Ratnam J, Ferreira RS, Jaishankar P, Zhao D, Arkin MR, McKerrow JH, Brinen LS, Renslo AR (2009). Novel non-peptidic vinylsulfones targeting the S2 and S3 subsites of parasite cysteine proteases. Bioorg Med Chem Lett.

[16] Sajid M, Robertson SA, Brinen LS, McKerrow JH (2011). Cruzain : the path from target validation to the clinic. Adv Exp Med Biol.

[17] Choe Y, Brinen LS, Price MS, Engel JC, Lange M, Grisostomi C, Weston SG, Pallai PV, Cheng H, Hardy LW, Hartsough DS, McMakin M, Tilton RF, Baldino CM, Craik CS (2005). Development of alpha-keto-based inhibitors of cruzain, a cysteine protease implicated in Chagas disease. Bioorg Med Chem.

[18] Huang L, Brinen LS, Ellman JA (2003). Crystal structures of reversible ketone-based inhibitors of the cysteine protease cruzain. Bioorg Med Chem.

[19] Huang L, Lee A, Ellman JA (2002). Identification of potent and selective mechanism-based inhibitors of the cysteine protease cruzain using solid-phase parallel synthesis. J Med Chem.

[20] Dimasi JA (2001). Risks in new drug development: approval success rates for investigational drugs. Clin Pharmacol Ther.

[21] Dolinsky TJ, Czodrowski P, Li H, Nielsen JE, Jensen JH, Klebe G, Baker NA (2007). PDB2PQR: expanding and upgrading automated preparation of biomolecular structures for molecular simulations. Nucleic Acids Res.

[22] Dolinsky TJ, Nielsen JE, McCammon JA, Baker NA (2004). PDB2PQR: an automated pipeline for the setup of Poisson-Boltzmann electrostatics calculations. Nucleic Acids Res.

[23] Duschak VG, Couto AS (2009). Cruzipain, the major cysteine protease of *Trypanosoma cruzi*: a sulfated glycoprotein antigen as relevant candidate for vaccine development and drug target. A review. Curr Med Chem.

[24] Krammer A, Kirchhoff PD, Jiang X, Venkatachalam CM, Waldman M (2005). LigScore: a novel scoring function for predicting binding affinities. J Mol Graph Model.

[25] Gehlhaar DK, Verkhivker GM, Rejto PA, Sherman CJ, Fogel DB, Fogel LJ, Freer ST (1995). Molecular recognition of the inhibitor AG-1343 by HIV-1 protease: conformationally flexible docking by evolutionary programming. Chem Biol.

[26] Muegge I, Martin YC (1999). A general and fast scoring function for protein-ligand interactions: a simplified potential approach. J Med Chem.

[27] Muegge I (2006). PMF scoring revisited. J Med Chem.

[28] Durrant JD, Keranen H, Wilson BA, McCammon JA (2010). Computational identification of uncharacterized cruzain binding sites. PLoS Negl Trop Dis.

[29] Christen M, Hunenberger PH, Bakowies D, Baron R, Burgi R, Geerke DP, Heinz TN, Kastenholz MA, Krautler V, Oostenbrink C, Peter C, Trzesniak D, van Gunsteren WF (2005). The GROMOS software for biomolecular simulation: GROMOS05. J Comput Chem.

[30] Ferreira RS, Bryant C, Ang KK, McKerrow JH, Shoichet BK, Renslo AR (2009). Divergent modes of enzyme inhibition in a homologous structure-activity series. J Med Chem.

[31] Feng BY, Simeonov A, Jadhav A, Babaoglu K, Inglese J, Shoichet BK, Austin CP (2007). A high-throughput screen for aggregation-based inhibition in a large compound library. J Med Chem.

[32] McGovern SL, Helfand BT, Feng B, Shoichet BK (2003). A specific mechanism of nonspecific inhibition. J Med Chem.

[33] Sherman W, Day T, Jacobson MP, Friesner RA, Farid R (2006). Novel procedure for modeling ligand/receptor induced fit effects. J Med Chem.

[34] Sherman W, Beard HS, Farid R (2006). Use of an induced fit receptor structure in virtual screening. Chem Biol Drug Des.

[35] Chagas C (1909). New human trypanosomiasis. Morphology and life cycle of *Schyzotrypanum cruzi*, the cause of a new human disease. Mem Inst Oswaldo Cruz.

[36] Chagas C (1911). A new human disease. Summary of etiological and clinical studies. Mem Inst Oswaldo Cruz.

[37] Wilkinson SR, Taylor MC, Horn D, Kelly JM, Cheeseman I (2008). A mechanism for cross-resistance to nifurtimox and benznidazole in trypanosomes. Proc Natl Acad Sci USA.

[38] Lauria-Pires L, Braga MS, Vexenat AC, Nitz N, Simoes-Barbosa A, Tinoco DL, Teixeira AR (2000). Progressive chronic Chagas heart disease ten years after treatment with anti-*Trypanosoma cruzi* nitroderivatives. Am J Trop Med Hyg.

[39] Schames JR, Henchman RH, Siegel JS, Sotriffer CA, Ni H, McCammon JA (2004). Discovery of a novel binding trench in HIV integrase. J Med Chem.

[40] Lin JH, Perryman AL, Schames JR, McCammon JA (2002). Computational drug design accommodating receptor flexibility: the relaxed complex scheme. J Am Chem Soc.

[41] Amaro RE, Schnaufer A, Interthal H, Hol W, Stuart KD, McCammon JA (2008). Discovery of drug-like inhibitors of an essential RNA-editing ligase in *Trypanosoma brucei*. Proc Nat Acad Sci.

[42] Durrant JD, Hall L, Swift RV, Landon M, Schnaufer A, Amaro RE (2010). Novel naphthalene-based inhibitors of *Trypanosoma brucei* RNA editing ligase 1. PLoS Negl Trop Dis.

[43] Durrant JD, Cao R, Gorfe AA, Zhu W, Li J, Sankovsky A, Oldfield E, McCammon JA (2011). Non-bisphosphonate inhibitors of isoprenoid biosynthesis identified via computer-aided drug design. Chem Biol Drug Des.

[44] Durrant JD, Urbaniak MD, Ferguson MA, McCammon JA (2010). Computer-aided identification of *Trypanosoma brucei* uridine diphosphate galactose 4′-epimerase inhibitors: toward the development of novel therapies for African sleeping sickness. J Med Chem.

[45] Wang Y, Hess TN, Jones V, Zhou JZ, McNeil MR, Andrew McCammon J (2011). Novel inhibitors of *Mycobacterium tuberculosis* dTDP-6-deoxy-l-lyxo-4-hexulose reductase (RmlD) identified by virtual screening. Bioorg Med Chem Lett.

[46] Morris GM, Huey R, Olson AJ (2008). Using AutoDock for ligand-receptor docking. Curr Protoc Bioinformatics.

[47] Wang R, Wang S (2001). How does consensus scoring work for virtual library screening? An idealized computer experiment. J Chem Inf Comput Sci.

[48] Bar-Haim S, Aharon A, Ben-Moshe T, Marantz Y, Senderowitz H (2009). SeleX-CS: a new consensus scoring algorithm for hit discovery and lead optimization. J Chem Inf Model.

[49] Berman HM, Westbrook J, Feng Z, Gilliland G, Bhat TN, Weissig H, Shindyalov IN, Bourne PE (2000). The protein data bank. Nucleic Acids Res.

[50] Gillmor SA, Craik CS, Fletterick RJ (1997). Structural determinants of specificity in the cysteine protease cruzain. Protein Sci.

[51] Mott BT, Ferreira RS, Simeonov A, Jadhav A, Ang KK, Leister W, Shen M, Silveira JT, Doyle PS, Arkin MR, McKerrow JH, Inglese J, Austin CP, Thomas CJ, Shoichet BK, Maloney DJ (2010). Identification and optimization of inhibitors of *Trypanosomal cysteine* proteases: cruzain, rhodesain, and TbCatB. J Med Chem.

