# Supplementary material for: Novel Cruzain Inhibitors for the Treatment of Chagas’ Disease
Source: Chem Biol Drug Des. 2012 Sep;80(3):398–405. doi: 10.1111/j.1747-0285.2012.01416.x (PMC3503458; doi:10.1111/j.1747-0285.2012.01416.x)
Supplement: Appendix S1 — Description of experimental validation methods and assessment of non-specific inhibition via aggregation. [file cbdd0080-0398-sd1.doc]

**Supporting Information**

*Varying Conditions in Enzymatic Assays to Validate Cruzain Inhibition*

The *Trypanosoma cruzi* cysteine protease cruzain was expressed and purified according to reference (33). All compounds were obtained from the NCI/DTP Open Chemical Repository ([http://dtp.cancer.gov](http://dtp.cancer.gov/)) and serially diluted in DMSO to create final assay concentrations (FAC) of 1000 μM to 0.01 μM. Using the standard conditions, the best and second-best scoring compounds had initial IC_50_ values of 471 nM and 15 µM, respectively (Table S1).

The top 8 compounds that inhibited cruzain at 100 μM were selected for further validation. In all assays, a buffer of 100 mM sodium acetate (NaAc) at pH 5.5 was used, and the initiation of cruzain (0.4 nM FAC) enzymatic activity was measured by adding fluorogenic substrate (2.5 μM FAC) Z-Phe-Arg-amino-methyl-coumarin (Z-FR-AMC) following a five-minute incubation with the novel compound. To investigate detergent-dependent sensitivity, the IC_50_ values of each compound in the presence of two different detergents were determined. First, results using Triton X-100 (0.02%) as the detergent were compared to those obtained without detergent (Fig. S1, left panel) to assess the general effect of detergent on cruzain inhibition. Next, assay results using Triton X-100 (0.02%) and Tween (0.002%) were likewise obtained for those compounds that did not appear to be aggregators (Fig.S1, on the right, last three rows). In order to assess inhibition dependence on the reducing agent, β-mercaptoethanol (BME) was subsequently used in place of DTT in the standard protocol for the four most potent compounds (Fig.S1, right panel).

Cruzain inhibition by each compound was evaluated by measuring the increase in fluorescence (excitation wavelength = 355 nm, emission wavelength of 460 nm) for five minutes in a microtiter plate spectrofluorimeter (Molecular Devices, FlexStation). Percent inhibition was determined from the initial velocities with SoftMax Pro 5.4, and dose response curves created in Prism 4 (GraphPad, San Diego, USA) were used to calculate IC_50_ values. Initial curves (detergent vs. no detergent) were generated by measuring inhibition at seven to eight concentrations of the predicted inhibitor, and final curves (best of at least two independent experiments of the detergent and reducing agent tests) were derived by measuring inhibition at fourteen to fifteen different concentrations, with the exception of NSC 61610, which included only 8 concentration measurements.

*Dynamic Light Scattering*

DLS experiments, performed according to reference (33), suggested that NSC 61610 was an aggregating compound even at low concentrations, with particles detected at 0.5 μM in both potassium phosphate (KPi) and sodium acetate (NaAc) buffers. AmpC β-lactamase (AmpC) was used to test for compound inhibition *via* aggregation (as described in reference (56)). NSC 61610 showed 52% AmpC inhibition at 5 μM, 30% inhibition at 1.5 μM, and 14% inhibition at 0.5 μM. Inhibition was reversible by adding 0.01% Triton X. NSC 67436 and NSC 260594 form particles at 15 μM and 40 μM KPi, respectively, but do not significantly scatter light in NaAc (up to 50 μM and 75 μM, respectively). There was no inhibition of AmpC up to 50 μM for either NSC 67436 or NSC 260594. NCS 227186 forms particles at 100 μM in KPi and 20 μM in NaAc.
