# Supplementary figures and images for: Novel Cruzain Inhibitors for the Treatment of Chagas’ Disease
Source: Chem Biol Drug Des. 2012 Sep;80(3):398–405. doi: 10.1111/j.1747-0285.2012.01416.x (PMC3503458; doi:10.1111/j.1747-0285.2012.01416.x)

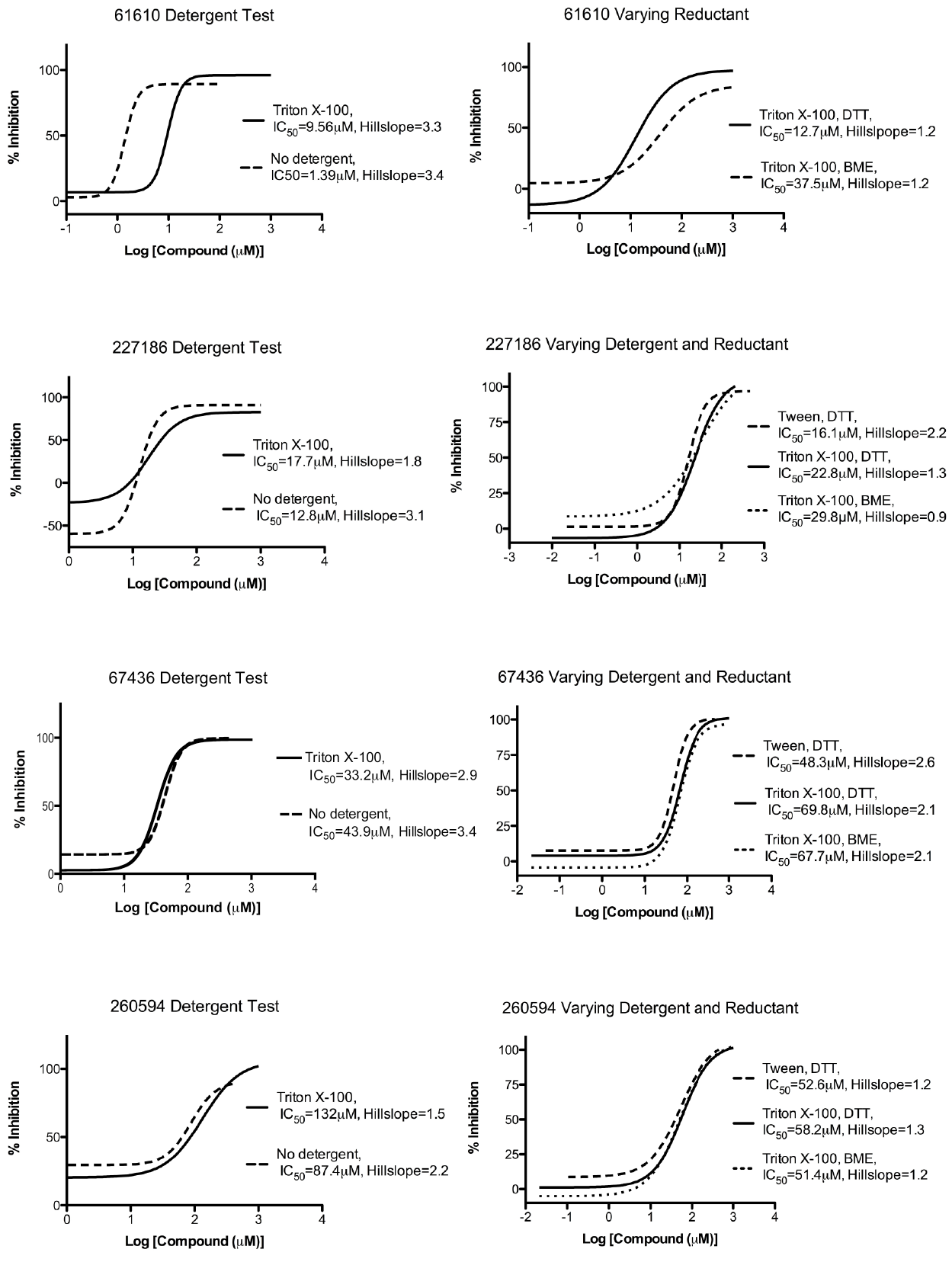

Supplement: Figure S1 — IC50 curves of each under various experimental conditions. [file cbdd0080-0398-sd2.png]
