# Supplementary material for: Novel Cruzain Inhibitors for the Treatment of Chagas’ Disease
Source: Chem Biol Drug Des. 2012 Sep;80(3):398–405. doi: 10.1111/j.1747-0285.2012.01416.x (PMC3503458; doi:10.1111/j.1747-0285.2012.01416.x)
Supplement: Table S1 — Preliminary experimental validationresults for compounds with IC50 values of100 μMor lower. [file cbdd0080-0398-sd3.doc]

Table S1: Preliminary experimental validation results for compounds with IC_50_ values of 100 μM or lower.

| Structure | ID | IC_50_ (µM) |
| --- | --- | --- |
|  | NSC 61610 | 0.471 |
|  | NSC 67436 | 15 |
|  | NSC 260594 | 39.1 |
|  | NSC 83950 | 73.6 |
|  | NSC 227186 | 94.9 |
|  | NSC 19803 | 98.2 |
|  | NSC 57670 | 98.3 |
|  | NSC 91529 | 100 |
